# The Americas are not on track to end TB: tuberculosis incidence analysis under the 2025 WHO goals

**DOI:** 10.3389/fpubh.2026.1830008

**Published:** 2026-05-28

**Authors:** Ángel Sebastián Rodríguez-Pazmiño, Solon Alberto Orlando, Camilo Cardenas Franco, Elsy Carvajal, Darwin Santiago Paredes, Greta Franco Sotomayor, Miguel Angel Garcia-Bereguian

**Affiliations:** 1 One Health Research Group, Universidad de Las Américas, Quito, Ecuador; 2 Universidad Espíritu Santo, Guayaquil, Ecuador; 3 Universidad Católica de Santiago de Guayaquil, Guayaquil, Ecuador; 4 Instituto Nacional de Salud Pública e Investigación, Guayaquil, Ecuador

**Keywords:** Americas, end TB strategy, surveillance, tuberculosis, WHO

Tuberculosis (TB) remains one of the leading infectious causes of death globally. Under the WHO *End TB Strategy*, adopted in 2014 and aligned with the Sustainable Development Goals (SDGs), countries committed to achieving a 50% reduction in TB incidence rate and a 75% reduction in TB deaths by 2025, compared with 2015 levels (1). However, the *Global TB Report 2025*, which presents data updated through 2024, indicates that global progress remains far off track. Since 2015, the global TB incidence rate has declined by only 12%, and TB deaths have decreased by 29%, both substantially below the 2025 milestones (2). Additionally, financing gaps persist following the commitments of the 2023 United Nations High-Level Meeting on TB, annual funding for TB research was expected to reach US$5 billion, yet only US$1.2 billion (24%) has been mobilized (2). While global trends are concerning, regional dynamics vary considerably. The Region of the Americas has historically had lower TB incidence than Africa and Southeast Asia, but recent reports suggest a worrisome reversal in several countries (3, 4). To assess regional progress, we analyzed trends in TB incidence rates across countries and dependent territories in the Americas, based on the most recent Global TB Report by WHO, available under the goals of the End TB Program.

The American continent comprises 58 geopolitical entities: 35 sovereign countries and 23 dependent territories. According to the WHO *Global TB Report* datasets for 2015 (baseline year) and 2024 (most recent year available), data were reported for 45 entities (for at least one of the two years). We excluded five entities (Anguilla, Bermuda, British Virgin Islands, Montserrat, and Barbados) because incidence data were missing for either 2015 or 2024. The final analysis included 40 entities: 3 in North America, 7 in Central America, 18 in the Caribbean, and 12 in South America. We extracted the WHO variable “e_inc_100k,” defined as the estimated TB incidence rate (all forms) per 100,000 population from the Global TB Report 2025. This variable aligns directly with the End TB Strategy milestone, which is defined as the reduction in the incidence rate relative to 2015. The percentage change between 2015 and 2024 was calculated for each entity, followed by analysis by subregion (country-by-country), and compared with the regional trend for the continent reported by the WHO (2024 vs. 2015).

According to the Global TB Report 2025, the net increase in TB incidence for the Region of the Americas was +13.0%. This represents a 63-percentage-point deviation from the 2025 target of a 50% reduction. In our analysis, we found that only six territories achieved or surpassed the 2025 target of a 50% reduction in incidence rate: Cuba (−85.3%), Saint Kitts and Nevis (−73.8%), Antigua and Barbuda (−71.1%), Grenada (−61.4%), Jamaica (−55.8%), and Dominica (−54.5%). Two additional territories were within 10 percentage points of the target: Haiti (−45.0%) and Curaçao (−41.7%). Nine other entities showed reductions ranging from −34.3% to −2.9%. In contrast, 22 countries and territories experienced increases in TB incidence rate, ranging from +3.0% to +2,650.0%. Entities exceeding the continental mean (+13.0%) included Brazil (+19.5%), Canada (+21.6%), and Guatemala (+21.7%). A group of countries and territories with higher increases included Sint Maarten (+30.2%), Uruguay (+43.1%), Aruba (+43.1%), Paraguay (+47.3%), Venezuela (+48.4%), Colombia (+48.5%), Trinidad and Tobago (+50.0%), Turks and Caicos Islands (+56.4%), Argentina (+57.1%), and Ecuador (+57.1%). But the most pronounced and worrying increases were observed in El Salvador (+118.9%), the Cayman Islands (+345.5%), Saint Lucia (+600.0%), Costa Rica (+675.0%), and Puerto Rico (+2,650.0). Table 1 summarizes the TB incidence results for each country and territory in the Americas.

**Table 1:** All results for tuberculosis incidence rate per 100,000 inhabitants (“e_inc_100k”) obtained from the Global TB Report for the years 2015, 2019, and 2024, with the calculation of the variation.

Country/dependent territory	Type of territory	Sub-region	e_inc_100k (2015)	e_inc_100k (2019)	e_inc_100k (2024)	Variation (%) 2015–2019	Variation (%) 2015–2024
Antigua and Barbuda^*^	Country	Caribbean	38	–	11	−100, 0	−71, 1
Aruba	Dependent territory	Caribbean	58	19	83	−67, 2	43, 1
Bahamas	Country	Caribbean	21	17	18	−19, 0	−14, 3
Cayman Islands	Dependent territory	Caribbean	11	59	49	436, 4	345, 5
Cuba	Country	Caribbean	75	68	11	−9, 3	−85, 3
CuraÃ§ao	Dependent territory	Caribbean	48	38	28	−20, 8	−41, 7
Dominica	Country	Caribbean	99	15	45	−84, 8	−54, 5
Dominican Republic	Country	Caribbean	59	45	51	−23, 7	−13, 6
Grenada	Country	Caribbean	44	26	17	−40, 9	−61, 4
Haiti	Country	Caribbean	318	239	175	−24, 8	−45
Jamaica	Country	Caribbean	52	35	23	−32, 7	−55, 8
Puerto Rico	Dependent territory	Caribbean	2	14	55	600, 0	2,650
Saint Kitts and Nevis	Country	Caribbean	42	43	11	2, 4	−73, 8
Saint Lucia	Country	Caribbean	8	34	56	325, 0	600
Saint Vincent and the Grenadines	Country	Caribbean	65	38	46	−41, 5	−29, 2
Sint Maarten (Dutch part)	Dependent territory	Caribbean	53	17	69	−67, 9	30, 2
Trinidad and Tobago	Country	Caribbean	18	19	27	5, 6	50
Turks and Caicos Islands	Dependent territory	Caribbean	55	93	86	69, 1	56, 4
Belize	Country	Central America	33	35	34	6, 1	3
Costa Rica	Country	Central America	12	99	93	725, 0	675
El Salvador	Country	Central America	53	63	116	18, 9	118, 9
Guatemala	Country	Central America	23	24	28	4, 3	21, 7
Honduras	Country	Central America	49	38	38	−22, 4	−22, 4
Nicaragua	Country	Central America	59	49	43	−16, 9	−27, 1
Panama	Country	Central America	58	39	63	−32, 8	8, 6
Canada	Country	North America	51	56	62	9, 8	21, 6
Mexico	Country	North America	25	26	28	4, 0	12
United States of America	Country	North America	33	29	32	−12, 1	−3
Argentina	Country	South America	28	33	44	17, 9	57, 1
Bolivia (Plurinational State of)	Country	South America	123	95	114	−22, 8	−7, 3
Brazil	Country	South America	41	45	49	9, 8	19, 5
Chile	Country	South America	18	19	19	5, 6	5, 6
Colombia	Country	South America	33	36	49	9, 1	48, 5
Ecuador	Country	South America	42	46	66	9, 5	57, 1
Guyana	Country	South America	102	80	67	−21, 6	−34, 3
Paraguay	Country	South America	55	60	81	9, 1	47, 3
Peru	Country	South America	131	131	139	0, 0	6, 1
Suriname	Country	South America	35	31	34	−11, 4	−2, 9
Uruguay	Country	South America	33	38	47	15, 2	42, 4
Venezuela (Bolivarian Republic of)	Country	South America	31	48	46	54, 8	48, 4

^*^This country was excluded from the sub-regional analysis because it had a missing data point.

To further explore the potential impact of the COVID-19 pandemic on TB incidence trends, we conducted an additional regional analysis comparing pre-pandemic (2019) and post-pandemic (2024) incidence rates with the 2015 baseline. Antigua and Barbuda was excluded from this analysis due to missing data, leaving 39 countries and territories included (Table 1). Regional TB incidence rates were calculated as population-weighted estimates using the formula: (sum of estimated TB cases/sum of population) × 100,000. Incidence values for the Americas and their subregions in 2015, 2019, and 2024 are presented in Supplementary Figure 1. At the continental level, TB incidence increased by 3.0% in 2019 and by 12.3% in 2024 relative to 2015 (based on our group of 39 countries and territories). At the subregional level, the Caribbean, Central America, North America, and South America showed changes of −19.9%, +13.4%, −6.3%, and +8.2% in 2019, respectively. By 2024, these variations had shifted to −41.1%, +34.2%, +3.0%, and +23.7%, respectively. Overall, the post-pandemic period was associated with a more pronounced deterioration in TB incidence trends than the pre-pandemic period, with the exception of the Caribbean region. However, importantly, most subregions already showed unfavorable trajectories before the COVID-19 pandemic, suggesting that the current failure to meet the End TB milestones cannot be attributed exclusively to pandemic-related disruptions. Among the analyzed subregions, the Caribbean showed the largest reduction relative to 2015 levels, although this finding should be interpreted cautiously given the influence of low baseline incidence and small-population territories.

According to the WHO Global Tuberculosis Report 2025, the Region of the Americas experienced the largest net increase in TB incidence between 2015 and 2024, with a rise of 13.0%, followed by the Western Pacific Region with a modest increase of 1.7%. In contrast, only two WHO regions achieved substantial reductions during this period: the European Region (−39%) and the African Region (−28%), although both remain short of the 2025 milestone targets. Overall, these trends indicate that the 2025 End TB milestones for a 50% reduction in TB incidence are being widely missed globally. The Americas show the most unfavorable trajectory among WHO regions, with the vast majority of countries far off track.

The heterogeneity observed across sub-regions is noteworthy. On the one hand, there is enormous variability across the Caribbean region. Eleven of 18 countries in the region show decreases ranging from −85.0% (Cuba) to −13.6% (Dominican Republic); however, the remaining seven show increases ranging from +30.2% to the outlier figure for Puerto Rico, at +2,650.0%. Central and North America follow similar trends. In the case of South America, following the same pattern as in 2023 (3), only three countries show a decrease: Suriname at −2.9%, Bolivia at −7.3%, and Guyana at −34.3%; the rest (9 out of 12) show increases ranging from +5.6% (Chile) to +57.1% (Argentina). Moreover, we call attention to the fact that extreme percentage increases observed in certain territories are largely driven by very low baseline incidence rates in 2015, where small absolute changes translate into disproportionately large proportional shifts. For example, Puerto Rico's increase from 2 to 55 cases per 100,000 population represents a +2,650.0% change, reflecting the mathematical sensitivity of percentage change when starting from near-zero values. Similar dynamics may partially explain sharp increases in Costa Rica (+675.0%) and Saint Lucia (+600.0%). These findings highlight the need for cautious interpretation of percentage changes in low-incidence settings, where modest absolute fluctuations or model-based revisions can lead to exaggerated relative trends. Furthermore, it is important to note that extreme proportional increases should be interpreted in the context of broader epidemiological and health system data of each territory.

It is important to note that the COVID-19 pandemic caused major disruptions in TB diagnosis, treatment, and prevention services worldwide (5). WHO documented sharp declines in TB case detection in 2020–2021, followed by partial recovery in subsequent years (6, 7). In some settings, increases in estimated incidence may reflect delayed detection, accumulated transmission during service disruptions, or improved post-pandemic surveillance. However, the magnitude of increases in several countries suggests that structural determinants are also contributing.

On the other hand, socioeconomic instability, migration, urban overcrowding, incarceration, and rising prevalence of comorbidities such as diabetes may have amplified TB vulnerability in parts of Latin America (8–11). TB is widely recognized as a disease strongly shaped by social determinants, including poverty and inequality (12). Economic downturns during and after the pandemic may have reversed prior gains in TB control. Additionally, financing gaps remain substantial. The persistent shortfall in TB research and program funding undermines the implementation of innovative diagnostics, the expansion of preventive therapy, and the development of shorter treatment regimens (2). Without accelerated investment, the End TB milestones may remain aspirational rather than achievable.

This study has several limitations that should be considered when interpreting the findings. First, the analysis was based on aggregated country-level estimates from the World Health Organization, which may be affected by differences in surveillance systems, case detection capacity, reporting quality, and diagnostic access across countries in the Americas. Furthermore, although these estimations represent the best available standardized metric for cross-country comparison, they are not direct measurements and rely on the good faith of the data each country presents, which may be influenced by a controversial political context (3). Second, because this was an ecological and descriptive analysis, the observed temporal changes cannot establish causal relationships between the COVID-19 pandemic and trends in tuberculosis incidence or mortality. Third, the use of selected time points and regional averages may mask important within-country heterogeneity and subnational dynamics. Finally, although the analysis identifies broad regional trends before and after the pandemic period, more sophisticated longitudinal approaches, including interrupted time-series analyses or country-specific modeling, would be necessary to better quantify the direct and indirect impacts of the pandemic on tuberculosis indicators in the region.

Despite these limitations, the findings raise concerns about the trajectory of TB control in the Americas. With the 2025 milestone imminent (results will be presented in the 2026 Global TB Report) and the 2035 SDG targets approaching, intensified multisectoral action will likely be required. However, the present analysis was not designed to directly determine the relative contributions of specific factors underlying the observed trends, and further studies incorporating population-level data, longitudinal approaches, and modeling analyses will be important for better understanding the drivers of the regional setbacks observed after the COVID-19 pandemic. If current patterns persist, the probability of achieving the 2035 elimination targets appears low for much of the continent. Reaching a 90% reduction would now require not only reversing recent increases but also sustaining historically unprecedented annual declines over the next decade. Modeling analyses have previously warned that even before the COVID-19 pandemic, global progress was insufficient to meet long-term End TB milestones (13, 14). The pandemic-related disruptions, combined with persistent social inequities and financing gaps, have further widened this gap (2, 14). Future research should also explore the influence of social determinants, healthcare disruptions, financing gaps, and diagnostic access on country-specific TB trajectories in the Americas. Without accelerated investment, strengthened health systems, expanded preventive therapy, and structural interventions to address the social determinants of TB, the 2035 targets may remain aspirational rather than attainable in the Americas. Urgent, region-specific strategies will be required to realign the continent with the End TB pathway.
