# Supplementary material for: The Americas are not on track to end TB: tuberculosis incidence analysis under the 2025 WHO goals
Source: Front Public Health. 2026 May 28;14:1830008. doi: 10.3389/fpubh.2026.1830008 (PMC13253538; doi:10.3389/fpubh.2026.1830008)
Supplement: Supplementary file 1 [file Supplementary_file_1.docx]

**Supplementary Figure 1.** Tuberculosis incidence rates (per 100,000 population) in the Americas and subregions in 2015, 2019, and 2024. The Caribbean showed the highest incidence rates across all evaluated years, although with a marked decline between 2015 and 2024. In contrast, Central America and South America exhibited increasing trends over the study period, while North America remained relatively stable. Overall, the incidence rate for the Region of the Americas increased from 40.7 cases per 100,000 population in 2015 to 45.7 in 2024.
